# Supplementary material for: A salivary GMC oxidoreductase of Manduca sexta re-arranges the green leaf volatile profile of its host plant
Source: Nat Commun. 2023 Jun 28;14:3666. doi: 10.1038/s41467-023-39353-0 (PMC10307781; doi:10.1038/s41467-023-39353-0)
Supplement: Supplementary file 1 — Supplementary Information [file 41467_2023_39353_MOESM1_ESM.pdf]

Supplementary Information

A salivary GMC oxidoreductase of *Manduca sexta* re-arranges the green leaf volatile profile of its host plant

Yu-Hsien Lin[1], Juliette Silven[1], Nicky Wybouw[2], Richard A. Fandino[3,7], Henk L. Dekker[4], Heiko Vogel[5], Yueh-Lung Wu[6], Chris de Koster[4†], Ewald Grosse-Wilde[3,8], Michel A. Haring[1], Robert C. Schuurink[1], Silke Allmann[1*]

[1] Green Life Sciences Research Cluster, Department of Plant Physiology, Swammerdam Institute for Life Sciences, University of Amsterdam, Amsterdam, Netherlands

[2] Terrestrial Ecology Unit, Department of Biology, Faculty of Sciences, Ghent University, Ghent, Belgium

[3] Department of Evolutionary Neuroethology, Max Planck Institute for Chemical Ecology, Jena, Germany

[4] Laboratory for Mass Spectrometry of Biomolecules, Swammerdam Institute for Life Sciences,

University of Amsterdam, Amsterdam, Netherlands

[5] Department of Insect Symbiosis, Max Planck Institute for Chemical Ecology, Jena, Germany

[6] Department of Entomology, National Taiwan University, Taipei, Taiwan

[7] Current Address: Department of Ecology & Evolutionary Biology, Cornell University, Ithaca, NY

[8] Current Address: EXTEMIT-K, Faculty of Forestry and Wood Sciences, Czech University of Life Sciences, 16500 Prague, Czech Republic

[†] Deceased in August 2019

[*] Correspondence and requests for materials should be addressed to S.A. (email: S.Allmann@uva.nl)

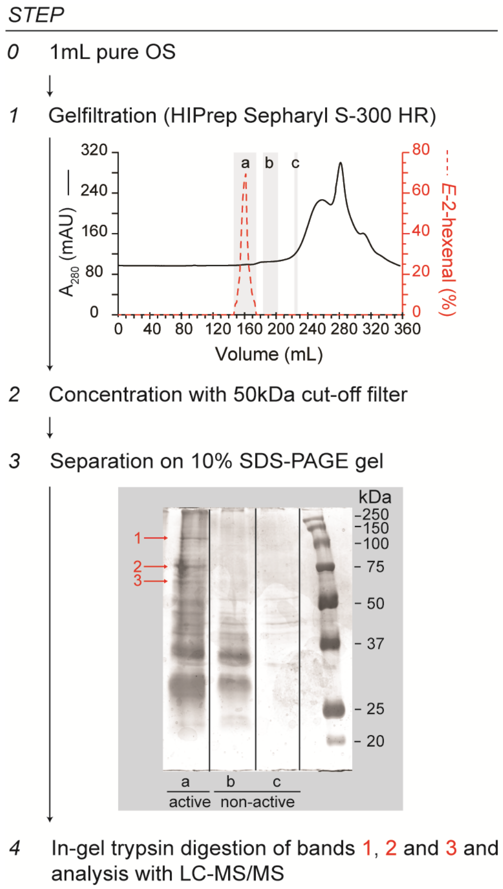

Supplementary Fig. 1. Partial purification of (3*Z*):(2*E*)-hexenal isomerase from *M. sexta* OS. 1 ml of pure *M. sexta* OS was diluted 1:1 (v/v) with Tris buffer (100mM NaSO4, 40mM Tris pH 9.0), applied to a HIPrep Sepharyl S-300 column and proteins were eluted isocratically (**Step 1**). Protein elution was continuously monitored by UV absorption at 280nm and is given in milli absorbance units (mAU, black line). The activity of each fraction was monitored using the SPME-guided *in vitro* assay and (3*Z*):(2*E*)-hexenal isomerase activity is given in % *E*-2-hexenal (red, dotted line). The pooled active fractions (grey box, **a**) and two pools of non-active fractions (grey boxes, **b** and **c**) were first each concentrated with centrifugal filters; proteins were subsequently precipitated with 80% acetone and re-suspended in MiliQ-water (**Step 2**). Samples were separated on a 10% SDS-PAGE gel and proteins were visualized by silver-staining (**Step 3**). The three most prominent bands that were solely present in the active sample (lane a, red arrows 1, 2 and 3) and absent in non-active samples (b and c) were cut-out from the gel; proteins were digested and extracted prior to mass spectrometric analysis by liquid chromatography-tandem mass spectrometry (LC/MS/MS) (**Step 4**)(n = 1 independent experiment).

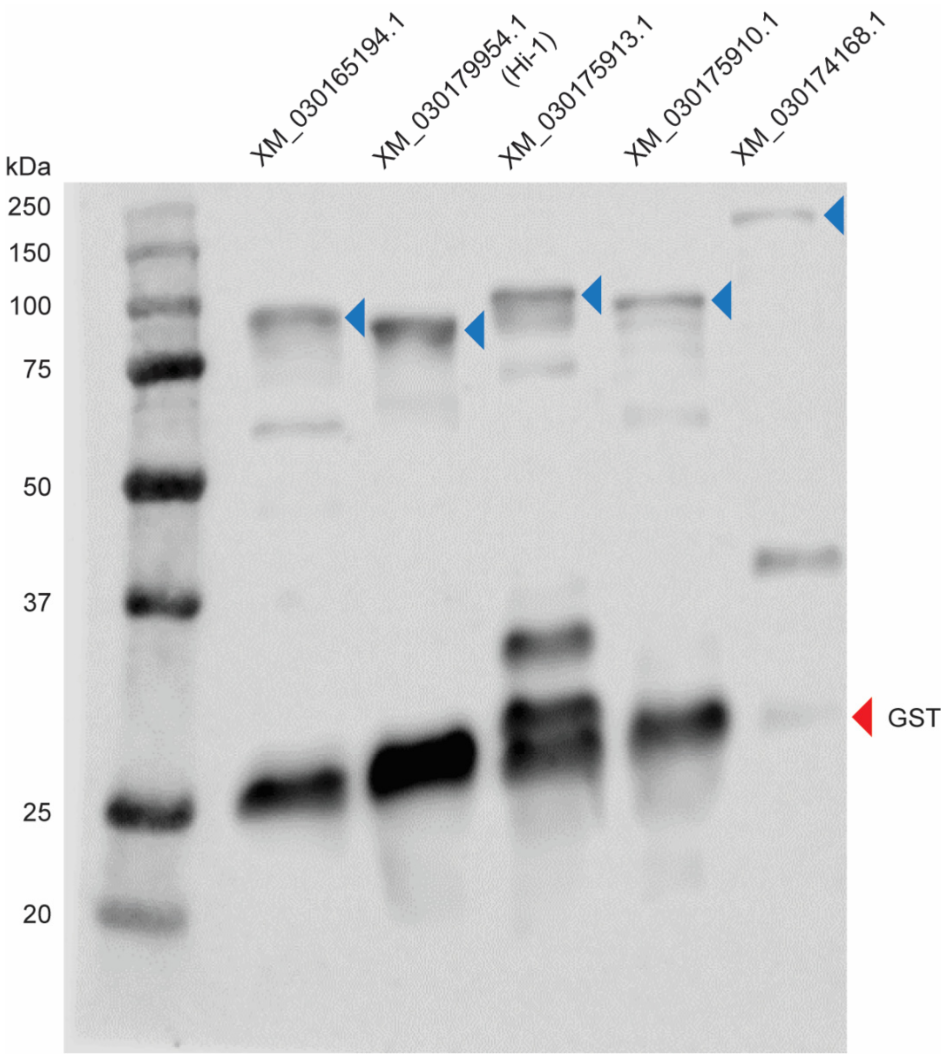

Supplementary Fig. 2. Full image of western blot shown in figure 2a. Purified GST-tagged recombinant protein of five candidate proteins (XM_030165194.1, XM_030179954.1, XM_030175913.1, XM_030175910.1 and XM_030174168.1) were visualized by western blot. Blue arrows indicate the position of each recombinant protein. Red arrow indicates position of GST.

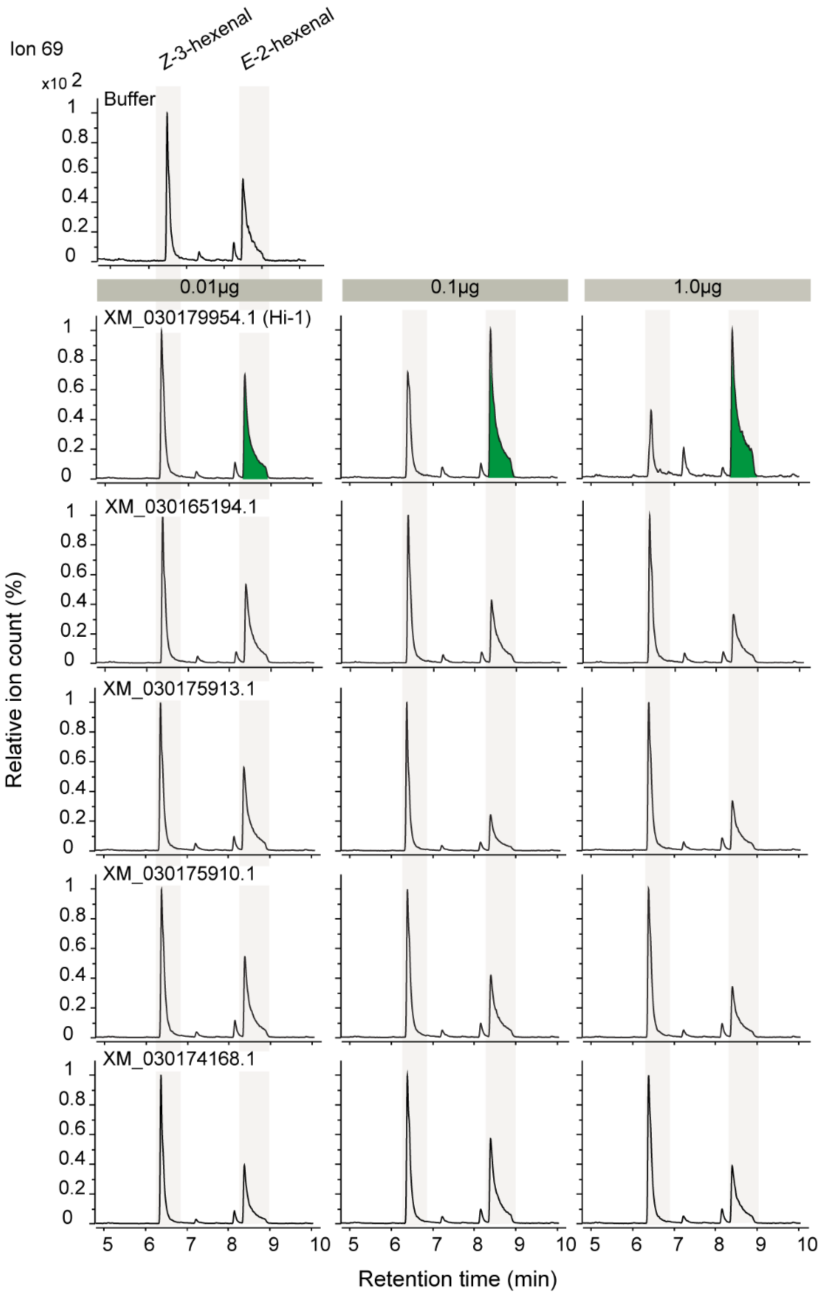

Supplementary Fig. 3. Representative extracted ion chromatograms (ion 69) of figure 2b. Hexenal isomerase activity of five candidate recombinant proteins with three different protein quantities (0.01, 0.1 or 1 µg) were determined by SPME-guided in vitro assay. Green highlight in Hi-1 indicates clear isomerase activity, converting *Z*-3-hexenal to *E*-2-hexenal.

	K_{cat} (s^{-1})	K_m (mM)	K_{cat}/K_m (s^{-1} mM^{-1})
Hi-1	121.04 ± 16.25	1.27 ± 0.45	114.87 ± 29.53

Supplementary Tab 1. Kinetic parameters of recombinant Hi-1 against Z3AL.

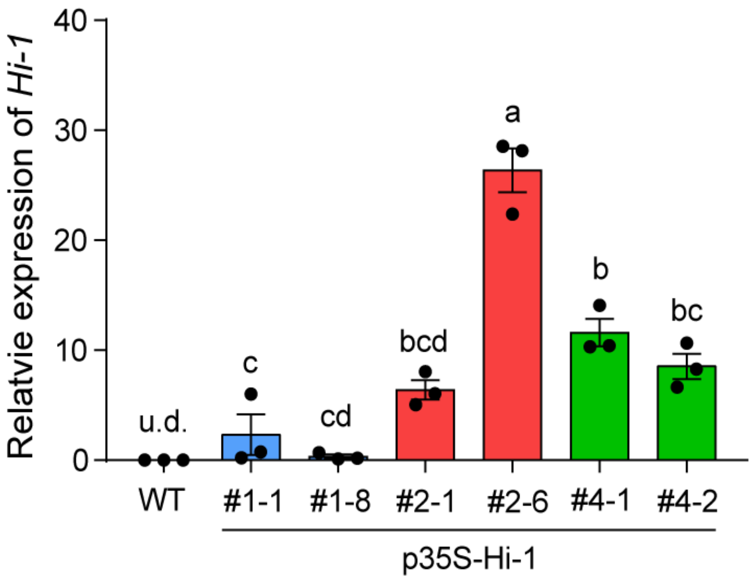

Supplementary Fig. 4. Transcript levels of *Manduca sexta*'s hexenal isomerase-1 (*Hi-1*) in stable expression lines of *Arabidopsis thaliana*. Measurement of *Hi-1* expression in transgenic *Arabidopsis* (Co1-0) T3-lines by qPCR. Whole shoot of 5-week-old plants were harvested. Expression level was determined by normalizing the *Hi-1* expression level to reference gene *sand* ($2^{\triangle CT}$ method). Different letters indicate significant differences ($p <$ 0.05) between groups (one-way ANOVA followed by Tukey HSD post-hoc analysis, $F_{5,12}$= 47.04, $p < 0.0001$, $n = 3$ biologically independent samples. Abbreviations: undetectable (u.d.), wildtype *Arabidopsis* (WT). Error bars are presented as mean values ± SEM.

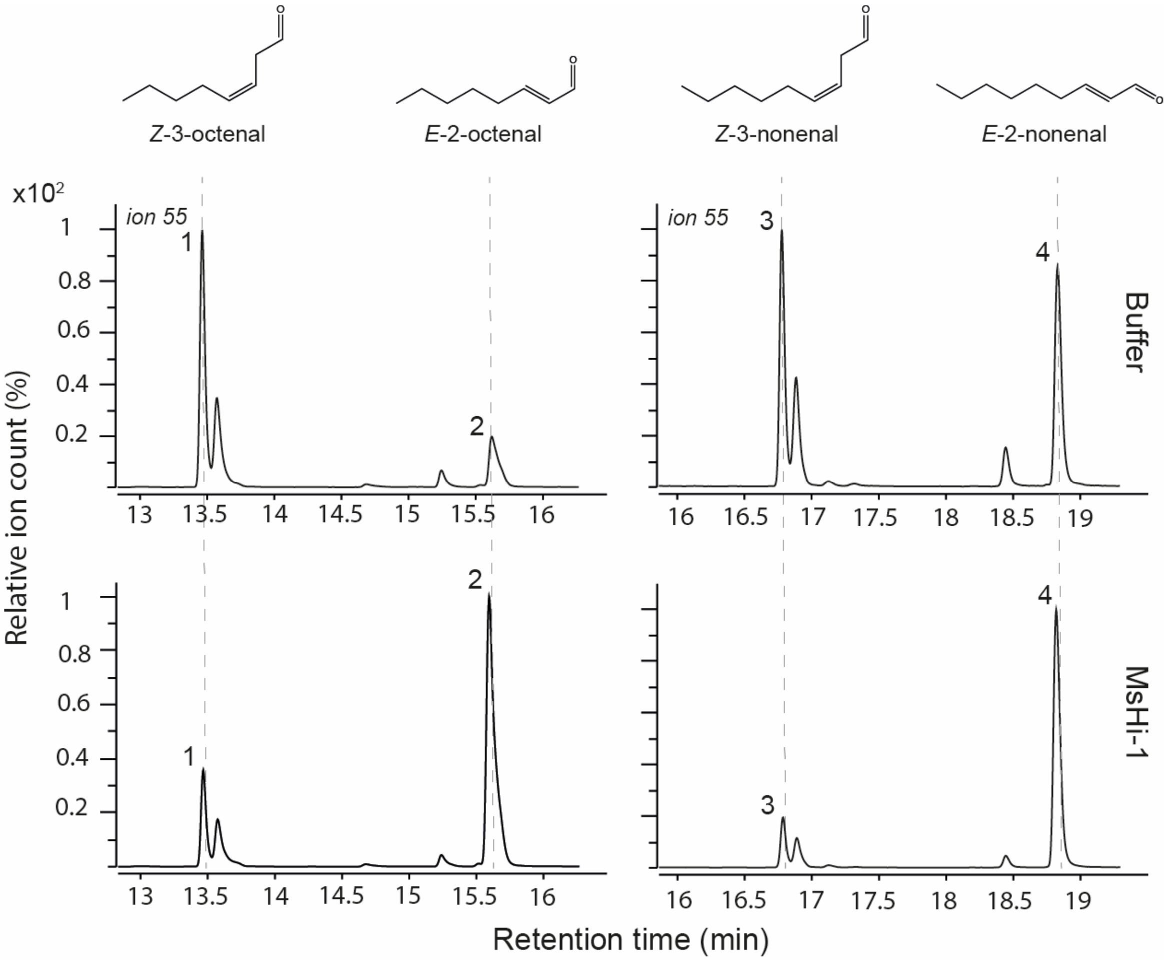

Supplementary Fig. 5. *M. sexta* Hi-1 utilizes *Z*-3-nonenal and *Z*-3-octenal as substrates. Representative extracted ion chromatograms (ion 55) of the SPME-guided assay to determine Hi activity of Hi-1 (0.1 µg) with two *Z*-3-alkenals (0.2 mM). Peaks 1 and 2 represent *Z*-3-octenal and *E*-2-octenal, respectively; peaks 3 and 4 represent *Z*-3-nonenal and *E*-2-nonenal, respectively. Three independent measurements were performed for both the (buffer) control and Hi-1.

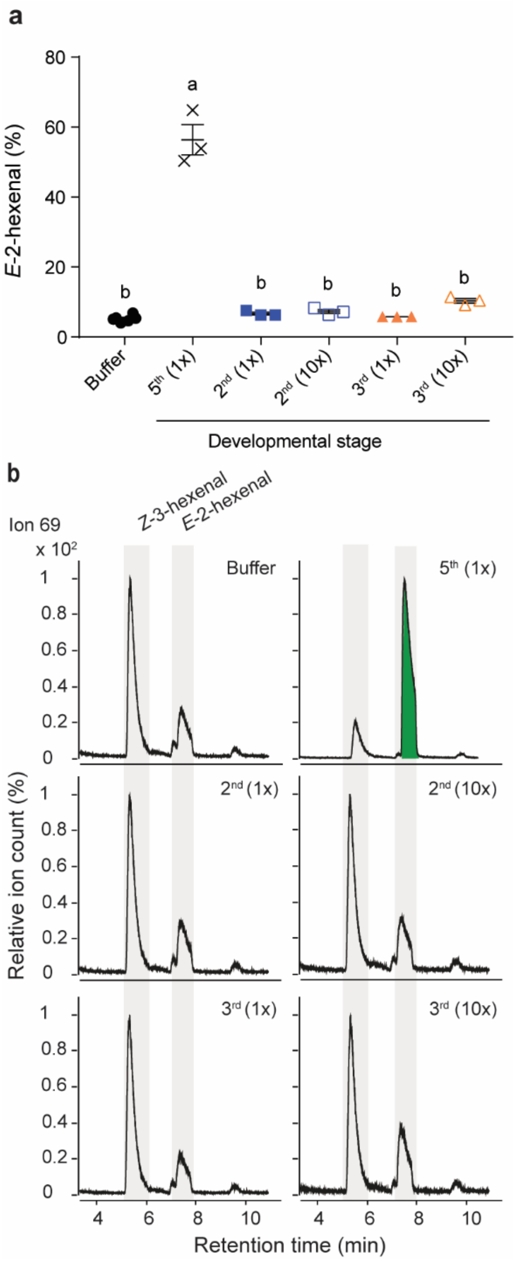

Supplementary Fig. 6. Hexenal isomerase activity of OS from 2nd and 3rd instar larvae with tenfold increased concentration. a 1X OS (0.5 µg total protein) or 10X OS (5 µg total protein) were incubated with Z-3-hexenal (1 mM), hexenal isomerase activity was determined by SPME-guided in vitro assay. The proportion of *E*-2-hexenal of total aldehydes (Z-3-hexenal + E-2-hexenal) was calculated. Different letters indicate significant differences ($p < 0.05$) between tissues (one-way ANOVA followed by Tukey HSD post-hoc analysis, $F_{5,15} = 155.3$, $p < 0.0001$, $n = 6$ for buffer, $n = 3$ for all OS samples (n represents biologically independent samples). **b** Representative extracted ion chromatograms (ion 69) as output of the SPME-guided assay. Green highlight in *E*-2-hexenal peak area indicates clear isomerase activity, converting *Z*-3-hexenal to *E*-2-hexenal. Error bars are presented as mean values ± SEM.

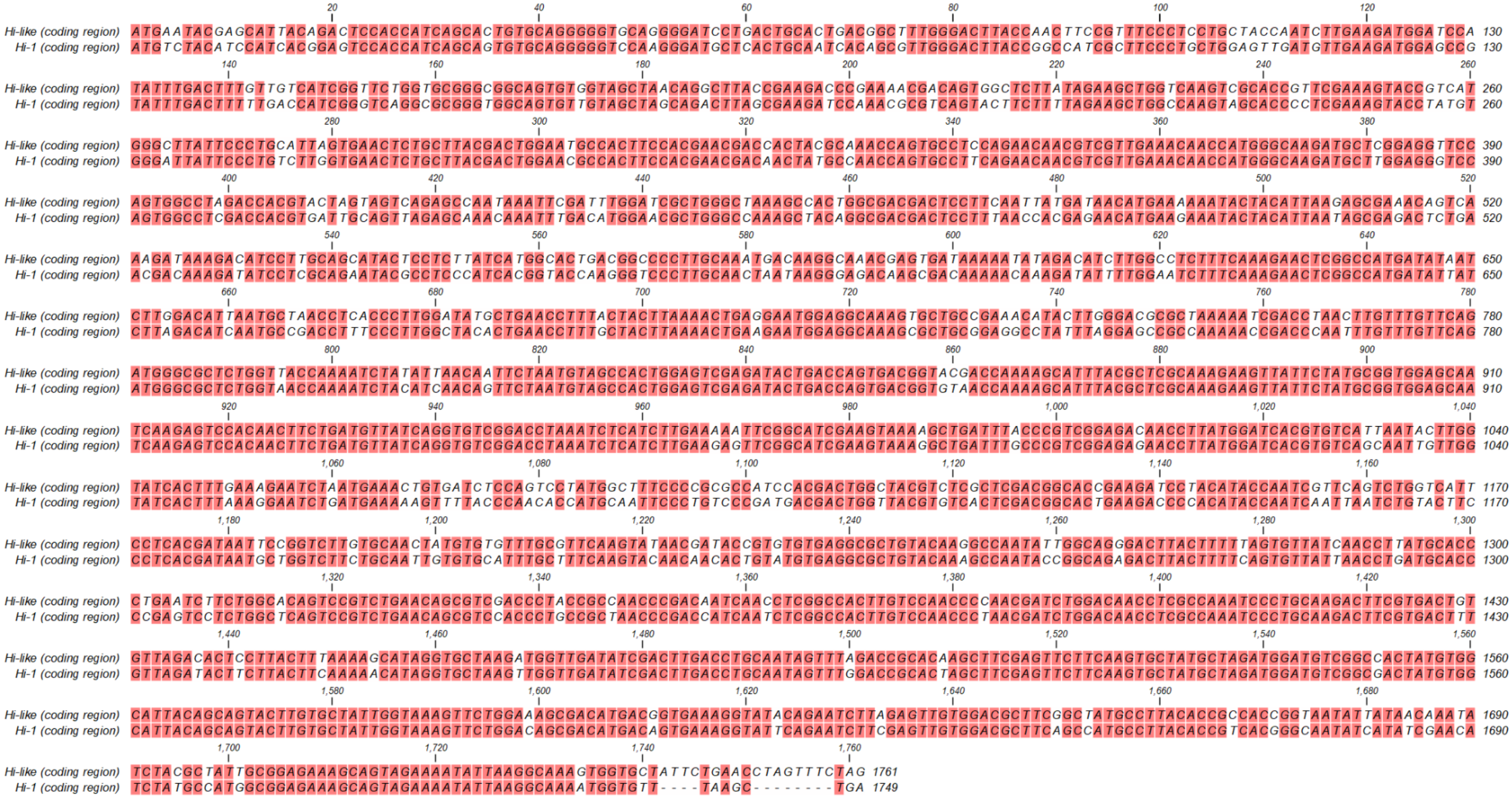

Supplementary Fig. 7. Alignment and sequence identity of *Hi-1* and *Hi-like* coding sequences. The coding sequences of Hi-1 (NCBI XM_030179954) and Hi-like (XM_030179956) were aligned by using CLC Main Workbench. The conserved nucleotides were highlighted by red color. Identity score was analyzed by using NCBI BLAST2; sequence identities:1479/1733(85%), Gaps:10/1733(0%).

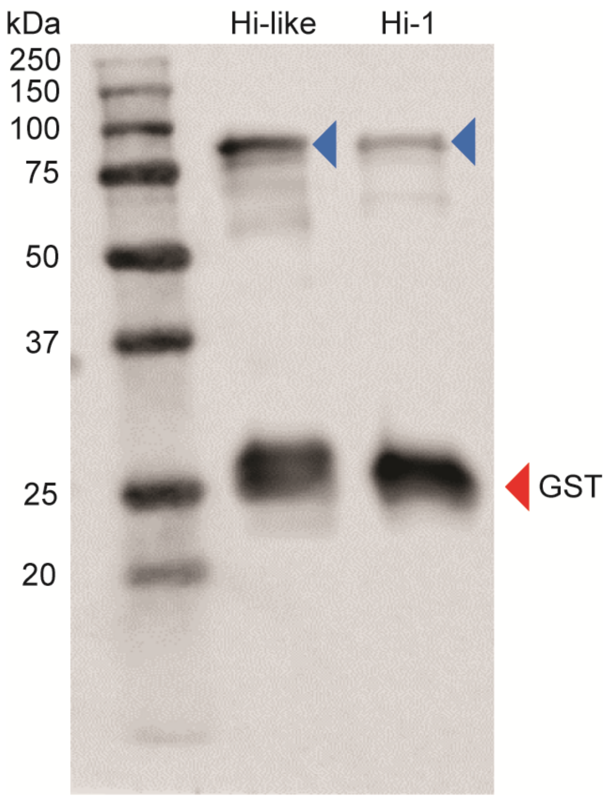

Supplementary Fig. 8. Full image of western blot shown in figure 5a. Purified GST-tagged recombinant proteins of Hi-like and Hi-1 were visualized on western blot. Blue arrows indicate the position of each recombinant protein. Red arrow indicates the position of GST.

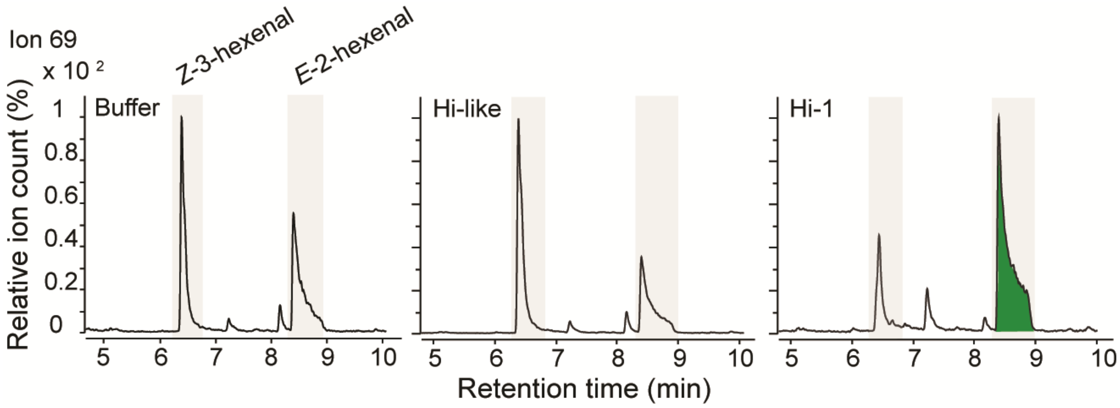

Supplementary Fig. 9. Representative extracted ion chromatograms (ion 69) of figure 5b as output of the SPME-guided assay. Green highlight in Hi-1 indicates clear isomerase activity converting *Z*-3-hexenal to *E*-2-hexenal.

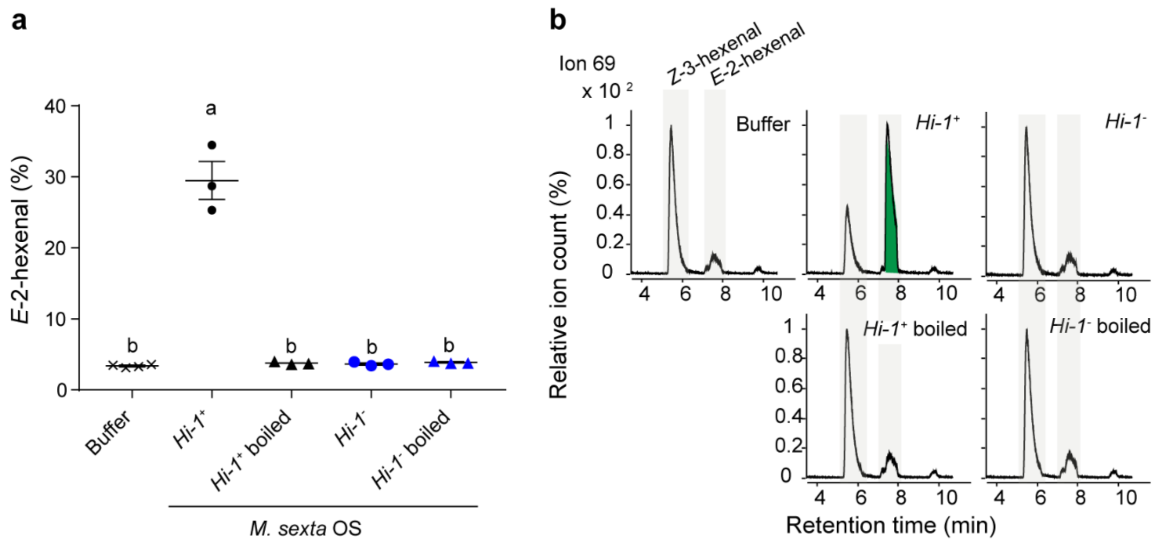

Supplementary Fig. 10. Hexenal isomerase activity of OS after heat treatment. **a** The OS samples (0.33 µg total protein) from wildtype (*Hi-1*$^{+}$ boiled) or homozygous mutant (*Hi-1*$^{-}$ boiled) were incubated at 95 ℃ for 5 min and hexenal isomerase activity was determined by SPME-guided in vitro assay with 200 mM of Z-3-hexenal as substrate. The proportion of *E*-2-hexenal of the total aldehydes (Z-3-hexenal + E-2-hexenal) was calculated. Different letters indicate significant differences ($p < 0.05$) between tissues (one-way ANOVA followed by Tukey HSD post-hoc analysis, $F_{4,11} = 103.4$, $p < 0.0001$, $n = 4$ for buffer, $n = 3$ for all OS samples, *n* represents biologically independent sample**s**). **b** Representative extracted ion chromatograms (ion 69) as output of the SPME-guided assay. Green highlight in *E*-2-hexenal peak area indicates clear isomerase activity, converting *Z*-3-hexenal to *E*-2-hexenal. Error bars are presented as mean values ± SEM.

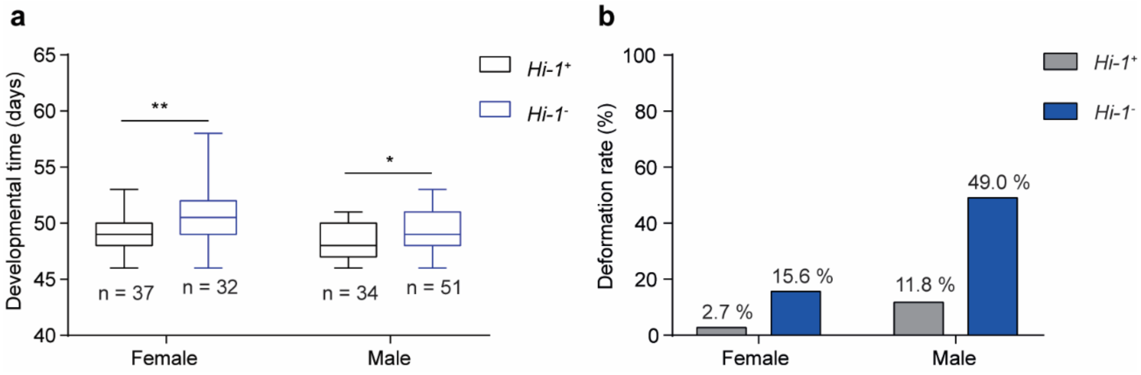

Supplementary Fig. 11. *Hi-1* mutant phenotypes under lower rearing temperature (24 °C). **a** Developmental time was recorded from neonate till newly emerged adult stage. Two-tailed t-test was performed comparing wildtype (*Hi-1*$^{+}$) and mutant (*Hi-1*$^{-}$) females (t = 3.34, df = 67, p = 0.0014) or males (t = 2.27, df = 83, p = 0.0258). For the box plot, the lower and upper hinges correspond to the 25th and 75th percentiles with the median as a line within interquartile range (IQR). The whiskers extend to the smallest and largest values within 1.5 times the IQR. **b** Proportions of deformed adults were recorded from one generation. *n* represents biologically independent samples. Error bars are presented as mean values ± SEM.

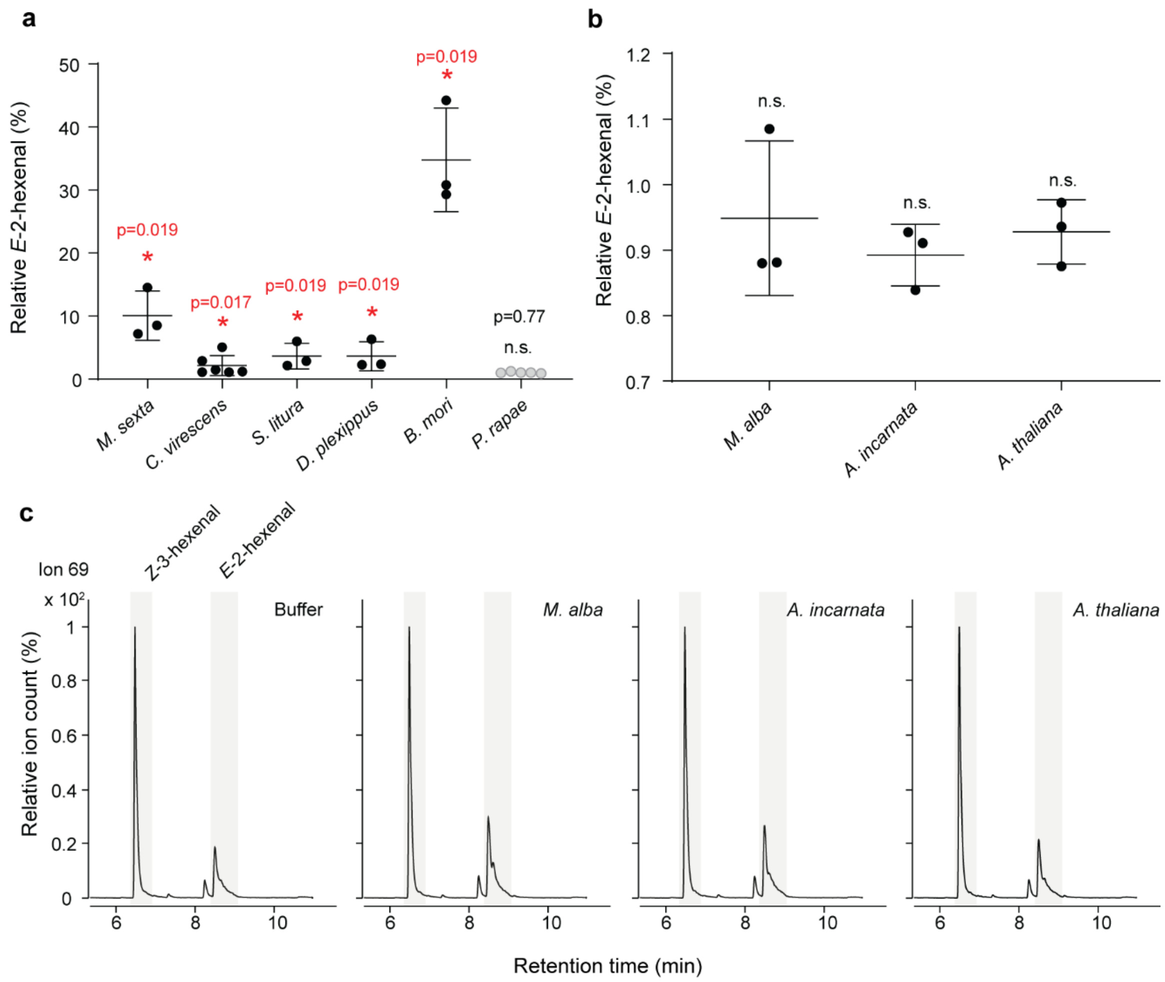

Supplementary Fig. 12. Hexenal isomerase activity in OS from six different lepidopterans and crude protein extract of their host plants. **a** OS (4 µg total protein) collected from 4th to 5th instar larvae, or **b** crude protein extract (4 µg total proteins) collected from leaves of three host plants were incubated with *Z*-3-hexenal (0.2 mM) and hexenal isomerase activity was determined by SPME-guided *in vitro* assay. The proportion of *E*-2-hexenal of the total aldehydes (*Z*-3-hexenal + *E*-2-hexenal) was calculated, and the value was further normalized to the buffer control. Asterisks indicate significant differences between buffer control and OS or plant extract (two-tailed Mann–Whitney U test, *p <0.05, n.s. not significant, n = 6 for *C. virescens*, n = 5 for *P. rapae*, n = 3 for buffer, *M. sexta*, *S. litura, D. plexippus, B. mori, M. alba, A. incarnata, A. thaliana*, n represents biologically independent samples). **c** Representative extracted ion chromatograms (ion 69) as output of the SPME-guided assay from the leaf's crude protein extracts with ten-fold concentration (40 µg total proteins). Error bars are presented as mean values ± SEM.

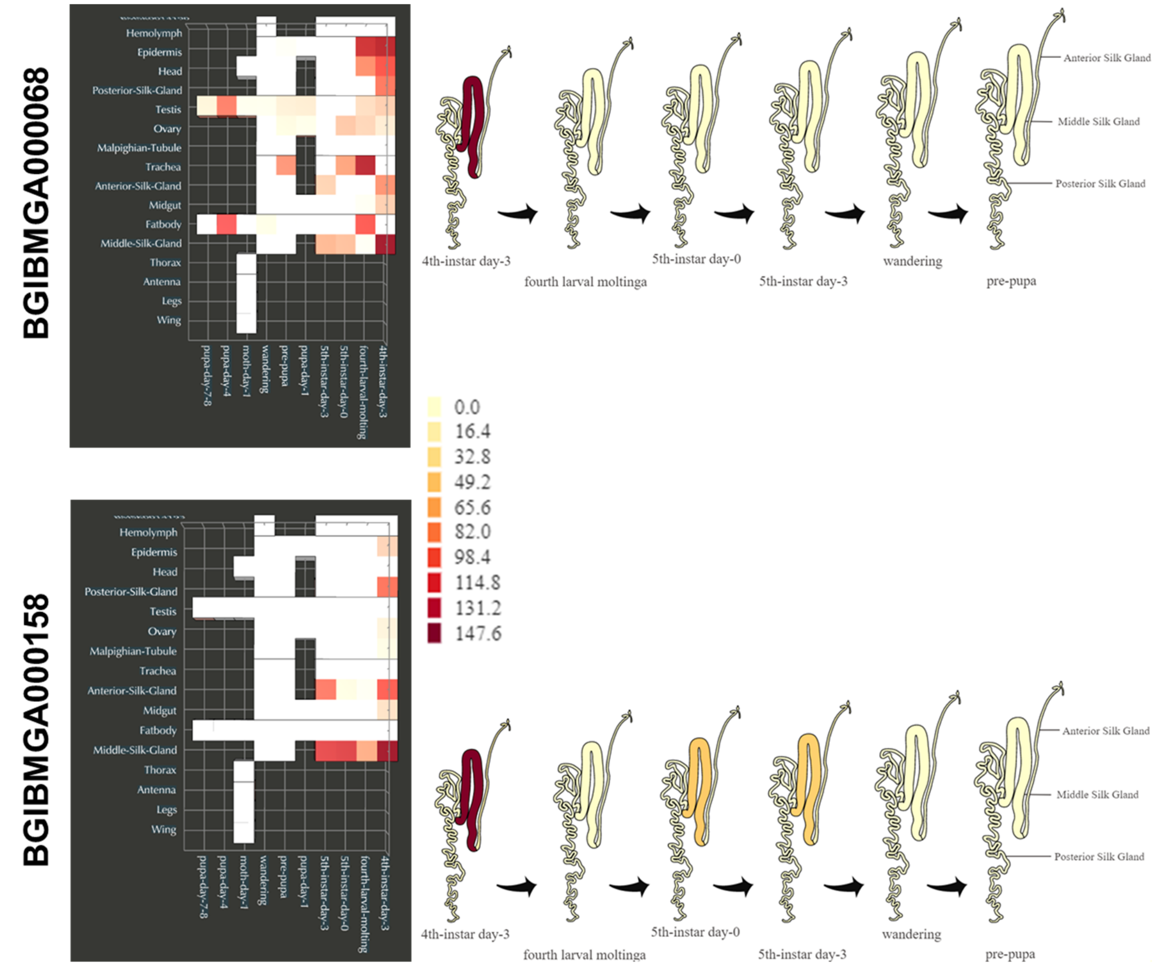

Supplementary Fig. 13. Spatial expression profiles of two *Bombyx mori* genes, BGIBMGA000068 and BGIBMGA000158. The data analysis and illustration are done by online tool, SilkDB 3.0[1].

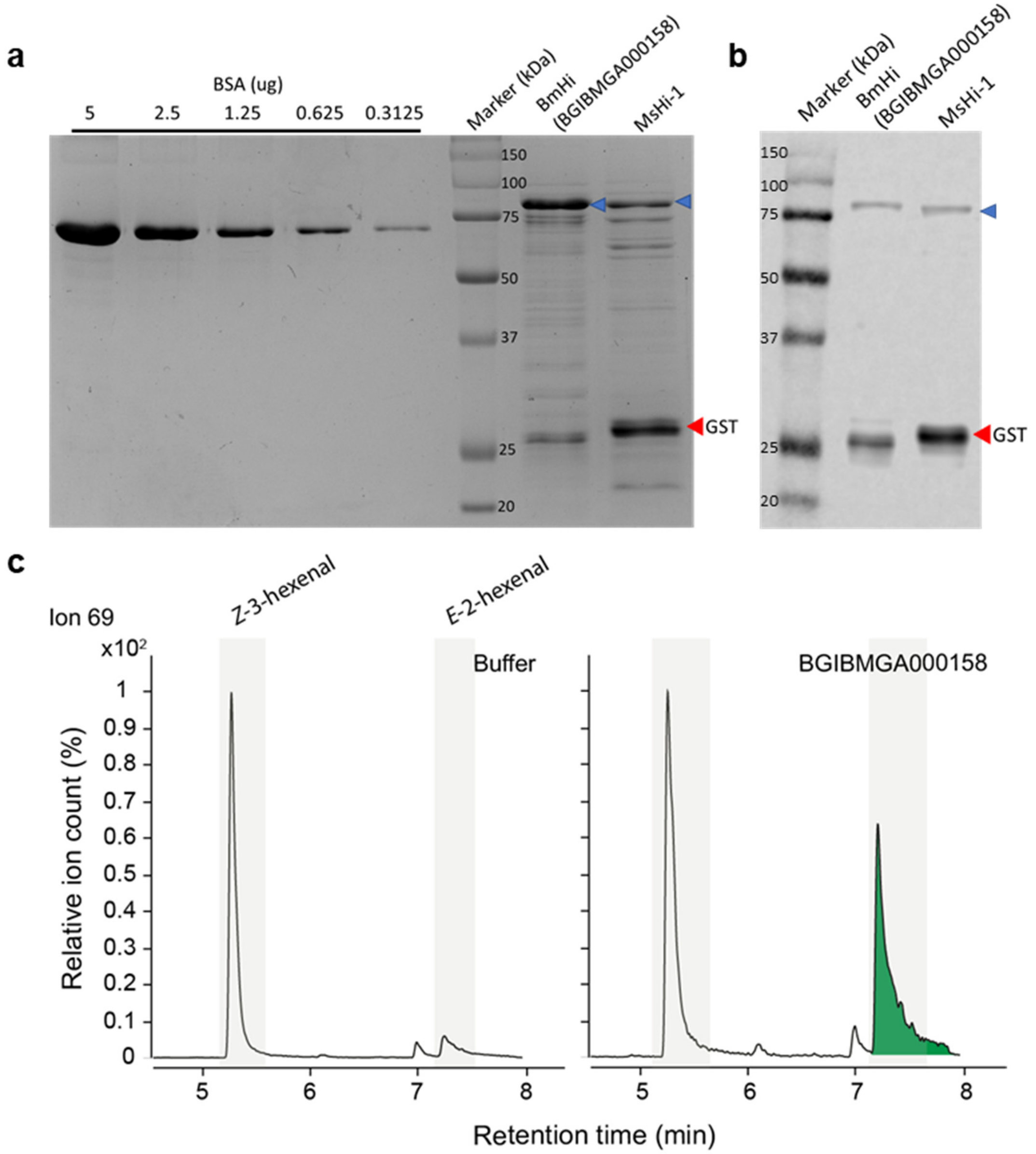

Supplementary Fig. 14. Recombinant BmHi production and measurement of hexenal isomerase activity. a Recombinant proteins were visualized by SDS-PAGE with coomassie blue staining. The relative intensity of bands between recombinant Hi and BSA standard was analyzed by ImageJ. Blue arrows indicate the position of BmHi. **b** Western blot imaging of BmHi and MsHi-1 (100 ng). Blue arrows indicate the position of each recombinant protein. **c** Representative extracted ion chromatograms (ion 69) of the SPME-guided assay to determine Hi activity of BmHi (1 μg) with Z3AL as substrate. Three independent measurements were performed for the buffer control and BmHi.

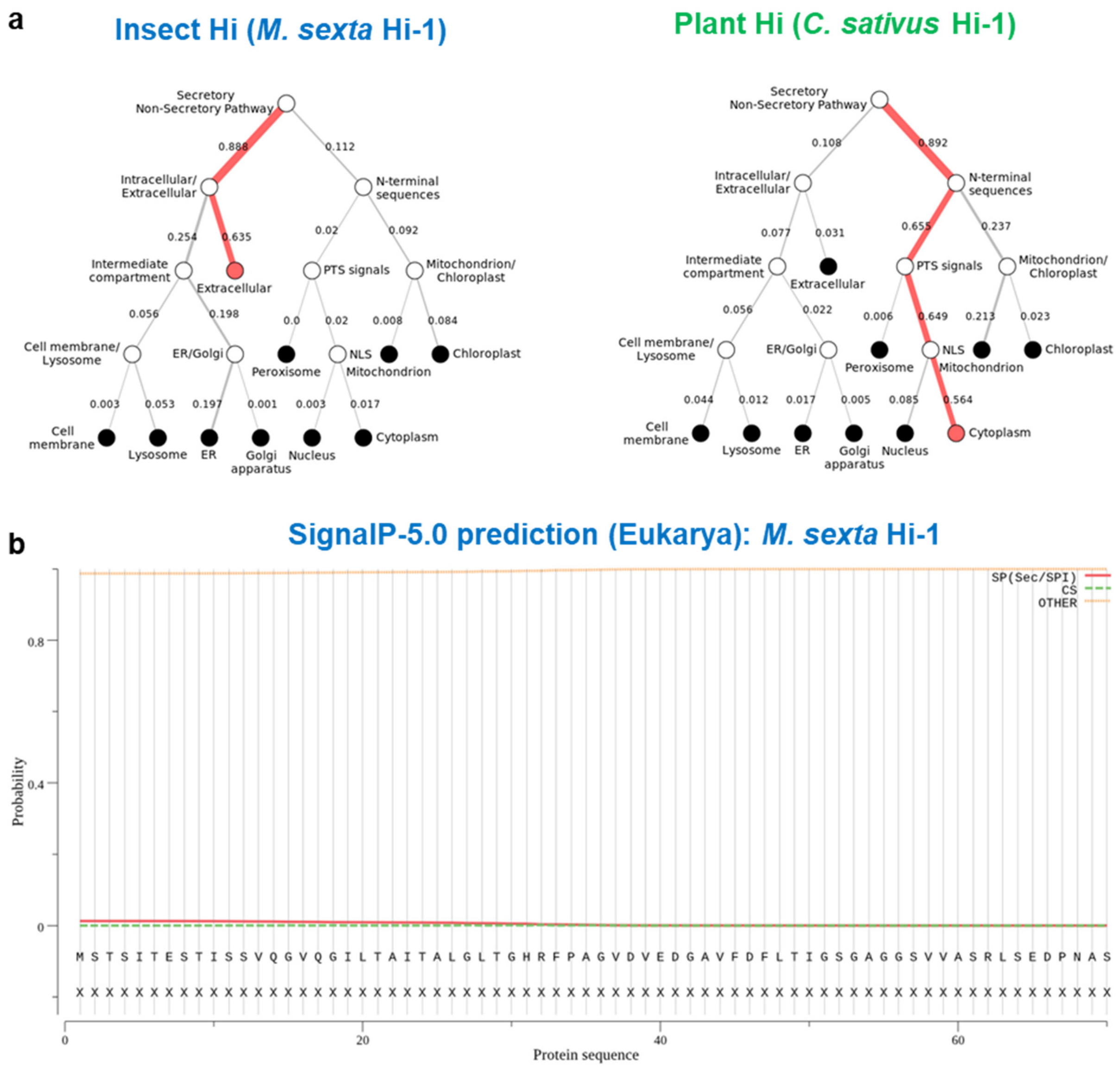

Supplementary Fig. 15. Hi-1 subcellular localization and signal peptide prediction in *M. sexta* Hi-1. **a** DeepLoc-1.0[2] was used to predict the subcellular localization. **b** SignalP-5.0[3] was used to predict the signal peptide sequence.

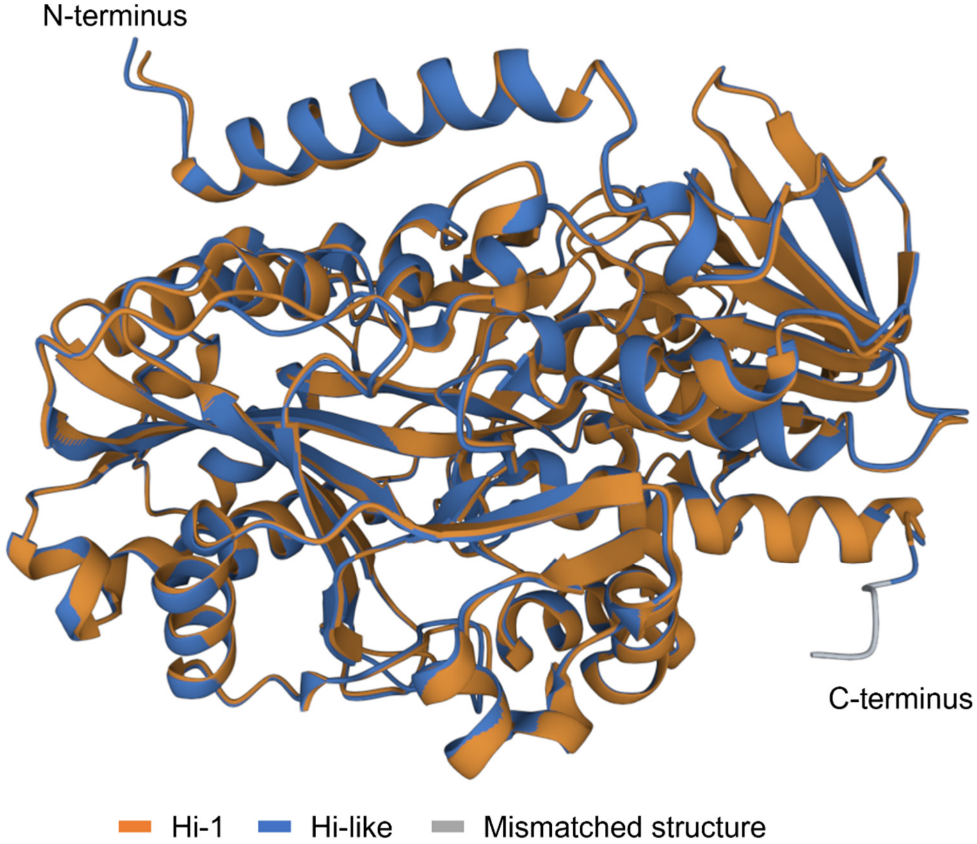

Supplementary Fig. 16. Pairwise alignment of Alphafold-predicted structures between *Manduca* Hi-1 and Hi-like. Top-ranked predicted structures were chosen for analysis. Pairwise structure alignment was performed on protein data bank (PDB) website, and jFATCAT-rigid method was chosen. Output scores: RMSD 0.48; TM-score 0.99; sequence identity 85%; reference coverage 100%; target coverage 99%.

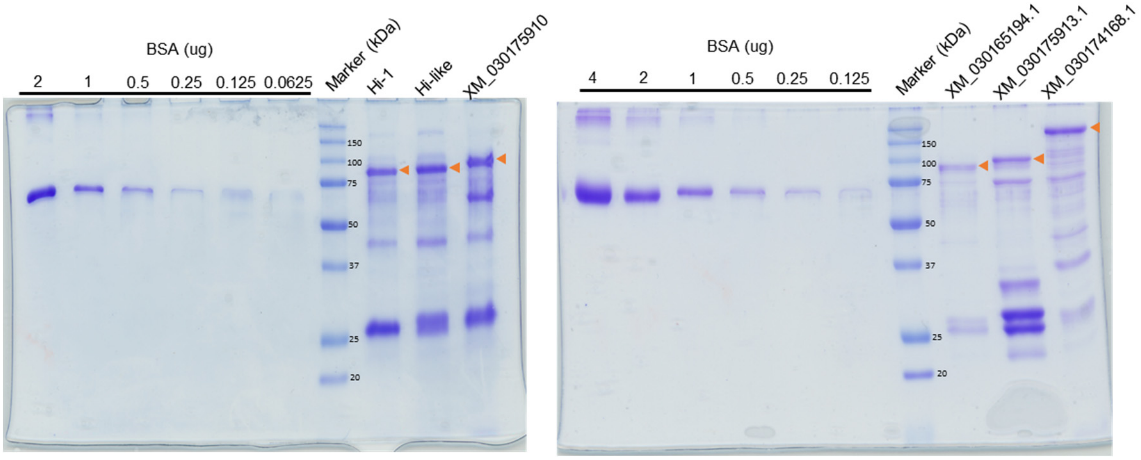

Supplementary Fig. 17. Quantification of recombinant proteins in SDS-PAGE using densitometry. The relative intensity of bands between recombinant proteins and BSA standard was analyzed by ImageJ. Arrows indicate the position of each recombinant protein (*n* = 1 independent experiment) .

***Z*-3-octenol** **   *Z*-3-octenal**

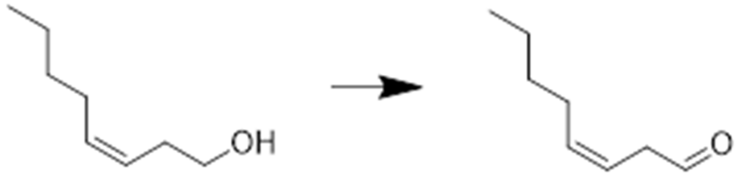

^{1}H 400 MHz NMR $CDCl_3$ [7.26 ppm]. 9.56 (t, 1H, J = 4Hz), 5.64-5.57 (m, 1H), 5.48-5.41 (m, 1H), 3.10 (dt, 2H, J = 4Hz), 1.96 (m, 2H), 1.26 (m, 4H), 0.80 (t, 3H, J = 8 Hz) ppm.

^{13}C 400 MHz NMR $CDCl_3$ [77.16 ppm]. 199.4, 135.2, 118.0, 42.4, 31.4, 27.2, 22.2, 13.8 ppm.

GC/MS calculated 124, found 124.

***Z*-3-nonenol** **   Z-3-nonenal**

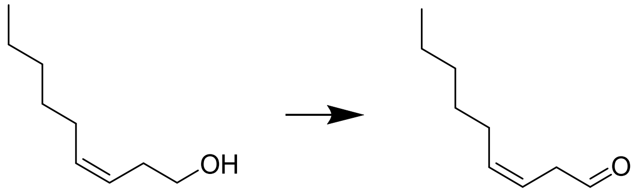

^{1}H 400 MHz NMR $CDCl_3$ [7.26 ppm]. 9.55 (t, 1H, J = 4 Hz), 5.64-5.57 (m, 1H), 5.48-5.41 (m, 1H), 3.08 (dt, 2H, J = 4Hz), 1.95 (m, 2H), 1.30-1.16 (m, 6H), 0.80 (m, 3H) ppm.

^{13}C 400 MHz NMR $CDCl_3$ [77.16 ppm]. 199.4, 135.3, 118.0, 42.4, 31.4, 28.9, 27.5, 22.4, 22.3, 13.9 ppm.

GC/MS calculated 140, found 140.

Supplementary Fig. 18. Synthesis of *Z*-3-octenal and *Z*-3-nonenal

Reference

1 Lu, F. *et al.* SilkDB 3.0: visualizing and exploring multiple levels of data for silkworm. *Nucleic Acids Research* **48**, D749-D755, doi:10.1093/nar/gkz919 (2019).

2 Almagro Armenteros, J. J., Sønderby, C. K., Sønderby, S. K., Nielsen, H. & Winther, O. DeepLoc: prediction of protein subcellular localization using deep learning. *Bioinformatics* **33**, 3387-3395, doi:10.1093/bioinformatics/btx431 (2017).

3 Almagro Armenteros, J. J. *et al.* SignalP 5.0 improves signal peptide predictions using deep neural networks. *Nature Biotechnology* **37**, 420-423, doi:10.1038/s41587-019-0036-z (2019).
