# Supplementary material for: A salivary GMC oxidoreductase of Manduca sexta re-arranges the green leaf volatile profile of its host plant
Source: Nat Commun. 2023 Jun 28;14:3666. doi: 10.1038/s41467-023-39353-0 (PMC10307781; doi:10.1038/s41467-023-39353-0)
Supplement: Supplementary file 3 — Description of additional supplementary files [file 41467_2023_39353_MOESM3_ESM.pdf]

<u>Description of additional supplementary files</u>

Supplementary Data 1: List of oral secretion candidate proteins from LC–MS/MS analysis.

Supplementary Data 2: Phylogeny tree with accession numbers.

Supplementary Data 3: List of primers and chemical reagents.
