# Supplementary figures and images for: A salivary GMC oxidoreductase of Manduca sexta re-arranges the green leaf volatile profile of its host plant
Source: Nat Commun. 2023 Jun 28;14:3666. doi: 10.1038/s41467-023-39353-0 (PMC10307781; doi:10.1038/s41467-023-39353-0)

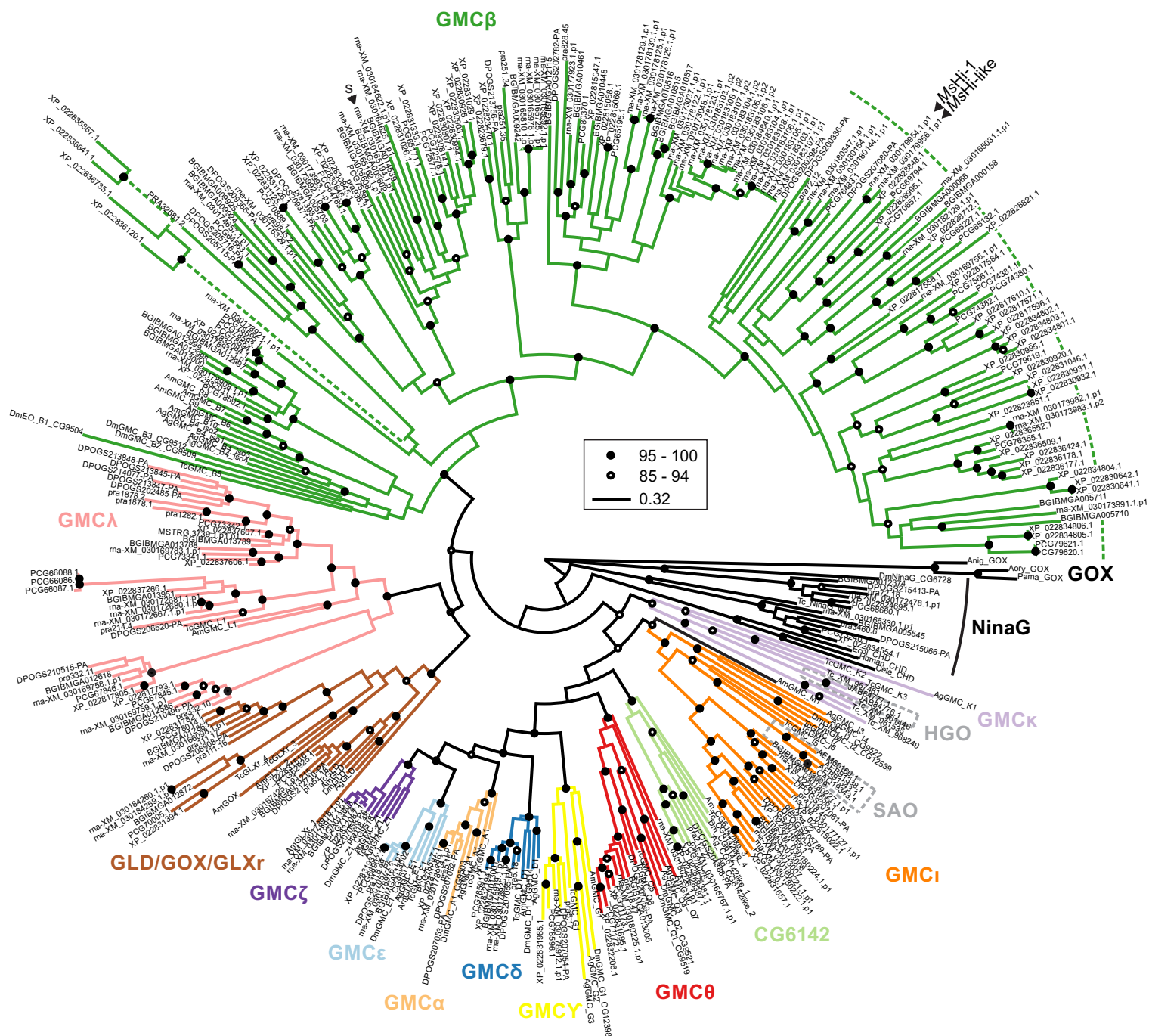

Supplement: Supplementary file 5 — Supplementary Data 2 [file 41467_2023_39353_MOESM5_ESM.pdf]
